# The Integral Boosting Effect of Selenium on the Secondary Metabolism of Higher Plants

**DOI:** 10.3390/plants11243432

**Published:** 2022-12-08

**Authors:** Liubov Skrypnik, Pavel Feduraev, Anton Golovin, Pavel Maslennikov, Tatiana Styran, Maria Antipina, Anastasiia Riabova, Dmitriy Katserov

**Affiliations:** MedBio Cluster, Immanuel Kant Baltic Federal University, 236041 Kaliningrad, Russia

**Keywords:** selenium, metabolic pathway, phenolic compounds, glucosinolates, alkaloids, redox metabolism, gene expression

## Abstract

Selenium is a micronutrient with a wide range of functions in animals, including humans, and in microorganisms such as microalgae. However, its role in plant metabolism remains ambiguous. Recent studies of Se supplementation showed that not only does it increase the content of the element itself, but also affects the accumulation of secondary metabolites in plants. The purpose of this review is to analyze and summarize the available data on the place of selenium in the secondary metabolism of plants and its effect on the accumulation of some plant metabolites (S- and N-containing secondary metabolites, terpenes, and phenolic compounds). In addition, possible molecular mechanisms and metabolic pathways underlying these effects are discussed. It should be noted that available data on the effect of Se on the accumulation of secondary metabolites are inconsistent and contradictory. According to some studies, selenium has a positive effect on the accumulation of certain metabolites, while other similar studies show a negative effect or no effect at all. The following aspects were identified as possible ways of regulating plant secondary metabolism by Se-supplementation: changes occurring in primary S/N metabolism, hormonal regulation, redox metabolism, as well as at the transcriptomic level of secondary metabolite biosynthesis. In all likelihood, the confusion in the results can be explained by other, more complex regulatory mechanisms in which selenium is involved and which affect the production of metabolites. Further study on the involvement of various forms of selenium in metabolic and signaling pathways is crucial for a deeper understanding of its role in growth, development, and health of plants, as well as the regulatory mechanisms behind them.

## 1. Introduction

Selenium (Se) is a metalloid with atomic number 34 and an atomic mass of 78.96. It is located in the 16th group of the periodic table—the chalcogen family. Selenium was discovered by Jöns J. Berzelius in 1817, but the study of its role in living organisms began after 1957 when Schwarz and Foltz showed that supplementing the diet with selenium prevents muscular dystrophy and liver cirrhosis in rats [1]. Selenium is chemically similar to sulfur, it can form covalent bonds with carbon, and is found in various organoselenium compounds, including selenoamino acids such as selenocysteine (SeCys) and selenomethionine (SeMet), as well as selenoproteins [2]. Selenium acts as the catalytic site of several selenoproteins, notably glutathione peroxidase (GSHPx), thioredoxin reductase, and iodothyronine deiodinase. Hence it is important for free radical scavenging, oxidative stress protection, and boosting the immune system [3].

The importance of Se was demonstrated for animals, humans, and some microorganisms, including microalgae [4,5,6,7,8,9]. However, in relation to plants, its essentiality is still controversial and has not been confirmed, despite the significant amount of experimental data showing that selenium can improve plant growth and development, as well as increase plant resistance to various abiotic and biotic stress factors [10,11,12,13]. Most researchers attribute the benefits of selenium to its effect on the antioxidant defense system of plants. Selenium was shown to stimulate the accumulation of low molecular weight antioxidants (ascorbate, glutathione, proline, phenolics) and the antioxidant enzymes activity (superoxide dismutase, catalase, ascorbate peroxidase, glutathione peroxidase, peroxidase). However, the exact mechanisms behind these improvements are complex and not fully understood [14,15,16,17,18].

Given that, selenium is essential for cellular function in many species and can be highly toxic, its presence is nuanced. It is generally found to be beneficial when present in trace amounts [2,3,19]. The most beneficial selenium concentrations, as well as the ways of its absorption, transport, and metabolism are largely determined by the plant species.

Based on the ability to accumulate selenium, plants are divided into three groups. The first group is selenium hyperaccumulators, capable of tolerating selenium concentrations in tissues in the range of 1000–15,000 μg Se per gram of dry weight (g^−1^ DW). They actively accumulate selenium up to these concentrations when grown on natural selenium-containing soils. This group includes some species of *Stanleya* (Brassicaceae) and *Astragalus* (Fabaceae).

The second group is selenium accumulators, capable of accumulating up to 1000 μg of Se g^−1^ DW and growing on both non-selenium and selenium-containing soils. The concentration of selenium in their tissues directly indicates the phytoavailability of selenium in the soil. Such plants are, for example, mustard (*Brassica juncea*), rapeseed (*Brassica napus*), broccoli (*Brassica oleracea* var. *italica*), arugula (*Eruca sativa*), and other members of the cruciferous family (*Cruciferae*), as well as plants of the *Allium* family (garlic, onion, leek).

The third group is selenium non-accumulators, which do not tolerate concentrations above 100 µg Se g^−1^ DW. This group includes such important crops as rice (*Oryza sativa),* wheat (*Triticum aestivum*), lettuce (*Lactuca sativa*), tobacco (*Nicotiana tabacum*), tomatoes (*Solanum lycopersicum*) and many others [2,19,20,21,22,23].

The hyperaccumulators are actively studied in order to identify the mechanisms of their tolerance to high concentrations of selenium, as well as their possible use for phytoremediation [24,25,26,27,28]. Both selenium-accumulating and non-accumulating plants are objects of biofortification research given their importance in the human diet [7,29,30]. Recent studies of selenium supplementation showed that not only does it increase the content of the element itself, but also affects the accumulation of other nutrients, secondary metabolites, and bioactive substances (for example, phenolics, glucosinolates, vitamin C and carotenoids) in plants [29,31]. That said, the mechanisms behind this improved accumulation of secondary metabolites are poorly researched, and the available data are inconsistent and contradictory.

Various review papers on selenium in plants were published in recent years. The main topics explored in these articles are the absorption, transport, and metabolism of selenium in plants [2,11,19,32,33,34], its role in the “soil-microorganisms-plant” system [35,36], its beneficial and toxic effects on plants [13,26,37], its part in increasing plant resistance to biotic and abiotic stresses [38,39,40,41,42], as well as the potential use of selenium in phytoremediation [25,26,28] and biofortification, including its effect on the accumulation of other nutrients [29,30,31,43,44,45,46]. However, this review article is focused on the generalization and analysis of the available data on the effect of selenium on the accumulation of secondary metabolites (sulphur and nitrogen-containing secondary metabolites, terpenes, and phenolics) in plants. In addition, possible molecular mechanisms and metabolic pathways underlying these effects will be discussed.

## 2. Selenium Uptake and Metabolism in Plants

**Figure 1:**
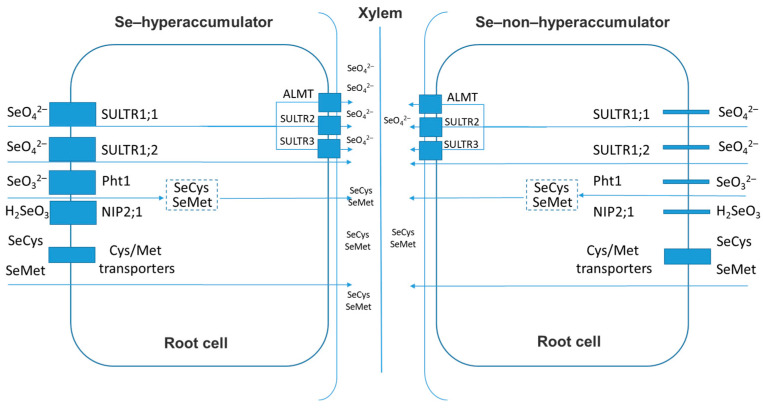
Pathways of Se uptake by plants classified as Se-hyperaccumulator and Se-non-hyperaccumulator.

Selenium absorption occurs in the rhizosphere solution through the root cells (Figure 1). Phytoavailable forms of selenium include selenates (SeO_4_^2−^), selenites (SeO_3_^2−^, HSeO^3−^, H_2_SeO_3_), and organoselenium compounds such as SeCys and SeMet, in contrast to selenides or colloidal elemental Se, which cannot be taken up by plants. In well-aerated oxygenated soils, selenate is the most abundant water-soluble form of selenium, while anaerobic soils with neutral to acidic pH are usually rich in selenite. In addition, soils usually contain small amounts of organoselenium compounds resulting from the decomposition of organic material and biological activity [47].

The intake of selenium, regardless of the form, leads to a change in the expression profile of plant cells directly responsible for the element absorption [48]. It should be noted that each of the forms induces a unique set of genes determining the success of absorption, transformation, and transfer of the compounds throughout the plant.

The transfer of selenate is carried out by symport transport systems, such as sulfate transporters (HAST), which include H^+^/sulfate symporters AtSULTR1;1 and AtSULTR1;2 [49]. Using Arabidopsis model plants (*Arabidopsis thaliana* [L.] Heynh.), it was shown that AtSULTR1;1 and AtSULTR1;2 catalyze the inflow of selenate into root cells. However, the degree of their involvement is directly correlated with the initial level of sulfur: when the level of S is high, most of the Se uptake is catalyzed by AtSULTR1;2, but if the level of S in the plant is too low for optimal growth, the contribution of AtSULTR1;1 increases instead. The expression of homologous genes in other Se non-accumulators is similar, while *AtSULTR1;1* and *AtSULTR1;2* in Se hyperaccumulators are constitutively expressed more strongly. As for Se-accumulators, they are characterized by a relatively high expression of both homologous genes and a preference for selenate, regardless of the initial level of sulfur [50]. This fact suggests that such plants are likely to show an increased content of selenium.

Another form of selenium, selenite, is taken up as HSeO_3_^−^ by the roots, then transferred by Pht1 (a member of the phosphate transporter family) and absorbed as H_2_SeO_3_ via aquaporins. It was shown that rice plants use namely aquaporin transport systems during the transfer of selenites, which leads to an increase in the expression of the *OsNIP2;1* gene. Selenite is rapidly converted to organoselenium compounds in the root, whereas selenate is immediately delivered to the xylem [51,52].

Organic forms of selenium, in particular SeCys and SeMet, are taken up by plant root cells via transporters that catalyze the uptake of Cys and Met, respectively [52]. However, the share of organoselenium compounds and selenites in overall selenium absorption is miniscule in comparison with selenates.

Investigating the pathways of intake and transformation of various forms of selenium is the key to understanding the role of this element in the metabolism of an autotrophic organism. There seems to be no single mechanism for its assimilation. This is indicated by the fact that selenium uptake and tolerance vary widely among different plant species (hyperaccumulation and non-accumulation described above).

Selenate absorbed by the root passes through the root cylinder and is loaded into the xylem for further transport to the shoot, while selenite requires rapid conversion into organoselenium compounds [52]. Then, a member of the ALMT family of transporters (organic acid transporters) is believed to load selenate into the xylem, although AtSULTR2;1, AtSULTR2;2 and AtSULTR3;5 are also involved in modulating this process by catalyzing the uptake of selenate into the parenchymal cells of pericycle and xylem [53,54]. Plastids are the central point for the conversion of selenate into organoselenium compounds [55]. The sulfate transporter AtSULTR3;1 is localized in the chloroplast membrane and seems to be capable of catalyzing the transport of selenate into plastids [56]. It is worth noting that the expression of key genes such as *APS* and several *SULTR* genes responsible for the assimilation of selenium into organic forms is regulated by the expression of microRNA (miRNA), such as miRNA395 [57].

**Figure 2:**
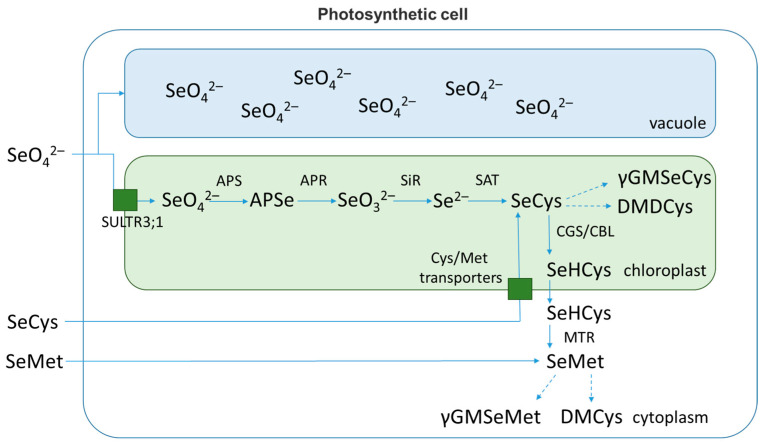
Metabolism of Se in plants.

Selenate is first activated by adenosine triphosphate sulfurylase (ATPS) to form adenosine 5^′^-phosphoselenate (APSe), which is then reduced to selenite by adenosine 5^′^-phosphosulfate reductase (APR) using reduced glutathione (GSH) as an electron donor (Figure 2) [58]. The conversion of selenate to selenite appears to be the rate-limiting step in the assimilation of Se into organic compounds [59]. This is indicated by the high level of organic selenium in the transgenic plant leaves with overexpression of genes encoding ATPS or APR [60,61]. A number of authors conclude that this stage affects the rate of selenium metabolism in non-hyperaccumulators more than in hyperaccumulators [20]. Afterward, selenite is reduced to selenide enzymatically by sulfite reductase, or non-enzymatically by reduced glutathione [59,62].

As already noted, the reduction of selenates to selenites is the rate-limiting step at the very first stage of selenium assimilation [63]. Selenium-hyperaccumulators can achieve this in at least three different ways: by replacing sulfate in the sulfate reduction pathway (reduction by ATP-sulphurylase/adenosine-5^′^-phosphosulfate reductase (APS)), by replacing nitrate in the nitrate pathway (microbial nitrate reductases exhibit ability to reduce selenate) or via specific selenate reductase [2,48,64].

For non-hyperaccumulators, there is strong evidence that selenate reduction occurs by replacing sulfate in the ATP-sulphurylase/APS-reductase system, which is the rate-limiting step in this case [48,63]. This transformation proceeds in two stages. Initially, ATP-sulphurylase catalyzes the interaction of ATP and selenate to form adenosine phosphoselenate. Then, under the action of APS-reductase, selenite is formed [63]. Interestingly, both reactions can occur in the cytosol as well as in the plastids of the cell due to the presence of isoforms of the enzymes indicated above [2,20]. According to other research, this stage occurs mainly in plastids and only a small part of selenium undergoes modification in the cytosol [2,63]. A number of articles mention the possible conversion of selenium into selenolipids or selenoglutathione via adenosine phosphoselenate [65].

The next step in selenium metabolism is the reduction of selenite to selenide. This step can proceed enzymatically in the presence of sulfite reductase. However, it is not completely clear whether this enzyme has selenite reductase activity [66]. Non-enzymatic interaction of selenite with reduced glutathione is also possible, resulting in the formation of selenodiglutathione GSSeSG. GSSeSG is then converted to GSSe, which is transformed into selenide by NADPH-dependent glutathione reductase [48,67].

The final step in the selenium assimilation is the conversion of selenide to selenocysteine (SeCys). This stage involves the conjugation of *O*-acetylserine with selenide by a multienzyme complex of cysteine synthase [63,66].

The resulting SeCys can undergo transformations in three known ways. One of them is the formation of elemental selenium by selencysteine lyase [68]. The second pathway involves the methylation of SeCys with the formation of methyl-SeCys by specific methyltransferase, which is most active in Se-hyperaccumulators [8,63]. In some hyperaccumulators, methyl-SeCys is additionally converted to the volatile dimethyl selenide compound [63]. In addition to dimethyl diselenide escaping into the atmosphere, a number of plants form γ-glutamylmethyl-SeCys, which acts as the main form of selenium storage in seeds. The third pathway consists of two stages. First, γ-homocystathionine synthase catalyzes the interaction of SeCys with O-phosphomoserine. As a result, selenocystathionine is formed, which is later degraded in the presence of cystathionine β-lyase to release selenohomocysteine. Both of these enzymes are localized in chloroplasts. The second stage of this pathway takes place in the cytosol, where methionine synthase initiates the formation of selenomethionine SeMet. In turn, SeMet can be additionally methylated to form methyl-SeMet, which is also converted into dimethyl selenide [63].

When there is an excess of selenium in the environment, non-accumulators tend to store selenate in the vacuoles of leaf cells, whereas hyperaccumulators modify inorganic selenium into less toxic organic forms or volatile compounds that are released as a result of gas exchange. Selenium assimilation leads to the formation of two amino acids, SeCys and SeMet, which can be incorporated into protein molecules.

## 3. Effect of Selenium on the Accumulation of Secondary Metabolites in Plants

### 3.1. Nitrogen-and Sulphur Containing Compounds

#### 3.1.1. Alkaloids

Plant alkaloids are an extensive group of secondary plant metabolites, structurally distinguished by the presence of a nitrogen atom in the molecule, outside the amide and peptide bonds. Natural precursors of alkaloids are derivatives of various metabolites formed during the glycolysis and shikimate pathways (mainly amino acids). In plants, alkaloids are allelopathic compounds whose function is closely related to seed formation and defense against pests [69].

There is little data on the effect of selenium on the biosynthesis of alkaloids, which indicates that this issue has not been sufficiently studied (Table 1).

Studies of strawberry (*Fragaria* × *ananassa*) biofortification with selenium showed that the addition of 100 µM of Na_2_SeO_4_ to the nutrient medium led to an increase in the concentration of the gramine alkaloid [70]. Another study investigating the effect of selenite-based foliar treatment, on the contrary, showed that selenium exposure leads to a decrease in the content of alkaloids in the seeds of white lupine (*Lupinus albus* L., variety Degas) [72].

In recent research involving foliar treatment of Iranian Borage plants with Na_2_SeO_3_ and Na_2_SeO_4_, it was found that the effect of selenium on the accumulation of alkaloids depended both on the selected form of selenium, its concentration, and the stage of flowering. The maximum increase in the content of alkaloids both in the leaves and in the petals of Iranian Borage plants was observed at the stages of initial flowering and termination of flowering, with a concentration of sodium selenate solution of 4 mg L^−1^. More specifically, when plants of the initial phase of flowering were treated with Na_2_SeO_4_, the content of alkaloids in flowers increased by 19.8% compared with the control sample. Spraying the plants with Na_2_SeO_3_ only led to an 8.5% alkaloid content increase [71]. To date, there is no clear understanding of the role of selenium in the biosynthesis of alkaloids. So far, it can be concluded that the role of selenium compounds in secondary metabolism reactions leading to the formation of alkaloids is most likely indirect. The relationship between the effect of selenium supplementation and the flowering stage suggests that this effect may be the result of various mechanisms, such as regulation of the hormonal signal or modulation of the thioredoxin system.

#### 3.1.2. Cyanogenic Glycosides

Cyanogenic glycosides, or α-hydroxynitrile glycosides, are a unique class of natural compounds containing a nitrile group, capable of releasing hydrogen cyanide (hydrocyanic acid) as a result of enzymatic decomposition. Similar to many other secondary metabolites, cyanogenic glycosides act as a defense against herbivores, releasing toxic hydrogen cyanide when tissue is damaged. Some plant species are lethal to humans due to their high content of cyanogenic glycosides [80]. The biosynthesis of cyanogenic glycosides is carried out using amino acids in a series of successive stages. These stages are catalyzed by two types of multifunctional enzymes—representatives of the cytochrome P450 family, and UDP-glucosyltransferase in combination with NADPH-dependent cytochrome P450 oxidoreductase [81].

The study of the effect of selenium treatment on the accumulation of cyanogenic glycosides is rarely performed (Table 1). This can be explained by various reasons. First, cyanogenic glycosides are not as common in plants as, for example, phenolic compounds. In addition, plants prone to the accumulation of cyanogenic glycosides are not suitable objects for selenium biofortification. For example, the order Brassicales is often used in studies of selenium biofortification. One of the widespread features of these species is the biosynthesis of glucosinates, another class of N-containing secondary metabolites. However, the joint presence of glucosinates and cyanogenic glycosides in the same plant is very rare [82]. A decrease in the accumulation of a cyanogenic glycoside, namely, 3,4-dihydroxymandelonitrile-β-glucoside, was observed in a study by Mimmo et al. [70], who carried out the non-targeted metabolic screening of strawberries during its biofortification with selenium.

#### 3.1.3. Glucosinolates

Glucosinolates are nitrogen- and sulfur-containing secondary metabolites and the so-called “molecular markers” of the Brassicales plants. The precursors in their biosynthesis are derivatives of various amino acids (methionine, valine, leucine, phenylalanine, tryptophan, etc.). Glucosinolates and the products of their degradation, primarily isocyanates, play a major role in the defense against phytophages [83,84].

Selenium is easily accumulated in *Brassica* species because Brassicaceae plants have the ability to metabolize Se into non-protein sulfurous amino acids, producing Se-methylselenocysteine (MeSeCys), γ-glutamyl-Se-methylselenocysteine (GGMeSeCys) and selenocystionine [85]. In this case, the close connection of selenium and sulfur metabolism explains the effect of selenium on the accumulation of glucosinolates, the biosynthesis of which is closely related to the metabolism of sulfur. Unlike the case of alkaloids and cyanogenic glycosides discussed above, there is a lot of research (including experimental) exploring the effect of selenium treatment on the accumulation of glucosinolates, but the results themselves are contradictory.

Broccoli (*Brassica oleracea* L. var. *italica*) is the experimental object most frequently used to study the effect of selenium biofortification on the accumulation of glucosinolates (Table 1). Tian et al. [73] treated the broccoli plants with sodium selenate solution at a concentration of 25 μM. It was found that selenium supplementation led to a decrease in the glucosinolates content in broccoli inflorescences and leaves, especially glucoraphanin. Notably, the suppression of glucosinolates accumulation was not associated with the absorption of sulfur by plants, the amount of which was not changed in the experiment. The inhibitory effect of selenium was explained by a decrease in the content of glucosinolates precursors—methionine and phenylalanine; expression of glucosinolates biosynthesis genes also significantly decreased after selenium exposure. In a study of the effect of various forms of selenium (100 μmol L^−1^ selenite and selenate) on the accumulation of glucosinolates in three varieties of broccoli sprouts, it was shown that selenium treatment did not lead to a significant change in the total content of glucosinolates (in two out of three varieties) but increased the content of sulforaphane and the activity of myrosinase (an enzyme catalyzing the hydrolysis of glucosinolates). For the third variety, a slight increase in the total content of glucosinolates was observed when plants were treated with selenate, accompanied by a decrease in the level of sulforaphane and myrosinase activity [74]. Similar results were obtained in a study on the joint effect of sulfur (as sulfate) and selenium (as selenite) on the metabolism of glucosinolates. Sulforaphane yield, myrosinase activity, and expression of the *MY* gene encoding myrosinase were significantly increased by the combined use of 4 mM S and 100 μM Se. In addition, the concentrations of glucoraphanin (a precursor of sulforaphane) and methionine (a substrate of glucoraphanin) did not change significantly after the application of Se [75]. In a study of the effect of various concentrations of selenate (0, 0.1, 0.2, 0.4, 0.8, and 1.6 mmol L^−1^) on the content of selenium, glucosinolates, and flavonoids in broccoli inflorescences, on the contrary, a sharp increase in the total glucosinolates content was found when using selenate at a concentration of 0.4 mM [76]. An increase in the total glucosinolates content in broccoli florets was determined in another study where plants were treated with selenium yeast and sodium selenite at concentrations of 0.1–1.6 mM [77].

The effect of selenium on the accumulation of glucosinolates was also studied on radishes (*Raphanus sativus*). Two experiments were carried out using different selenium treatments: foliar (0.5, 10, and 20 mg Se per plant) and hydroponics (0.5, 10, 20, or 40 μM), for 1 week. When using foliar treatment, the total level of glucosinolates in the roots was 35% higher compared to the control due to the increase in the level of glucoraphanin. When selenium was added to the nutrient medium, the level of glucosinolates in the roots did not change significantly. At the same time, a decrease in glucosinolates was observed in the leaves [78]. The addition of selenium to the nutrient medium (as selenate of 5–40 μM) during the hydroponic cultivation of two rocket species (*Eruca sativa* Mill. and *Diplotaxis tenuifolia*), a decrease in both the total content of glucosinolates and individual representatives of this class was observed compared to control plants [79].

Generally, the change in the level of glucosinolates in selenium-treated plants can be due to several reasons: changes in the content of amino acids that act as precursors in the biosynthesis of glucosinolates; changes in the expression of genes involved in it, or a change in the activity of myrosinase.

### 3.2. Terpenes

Terpenes are the largest and most diverse class of secondary metabolites. In plants, they act as informational and protective molecules used by plants for antagonistic and mutualistic interactions [86]. All terpenes in plants are produced from the five carbon precursors: dimethylallyl diphosphate (DMAPP) and its isomer isopentenyl diphosphate (IPP). Terpenes can be present as single unit molecules (hemiterpene (5C), mono-(C10), sesqui-(C15), di- (C20), sisters- (C25), tri- (C30), tetra- (C40)) or as polyterpenes, the number of units in which is more than eight and can reach 10^3–4^ [87]. General studies on the effect of selenium on the accumulation of terpene-like secondary metabolites show that the authors mainly evaluate the effect of selenium on the total accumulation of essential oils and their qualitative composition, as well as on the accumulation of tetraterpenes, namely carotenoids. Therefore, this section will focus on the effect of selenium on the accumulation of essential oils, the main components of which are mono- and sesquiterpenes, as well as carotenoids.

#### 3.2.1. Essential Oils

Essential oils are trivially thought of as oily liquids composed of volatile compounds that are released by physical action (such as pressing or distillation). Essential oils are complex mixtures of monoterpene and sesquiterpene hydrocarbons and their oxygen-containing derivatives (alcohols, aldehydes, ketones), as well as phenylpropanoids. In some cases, essential oils may include other chemical groups such as fatty acids, oxides, and sulfur derivatives [88]. Essential oil components are synthesized through three biosynthetic pathways: mevalonate, methylerythritol phosphate and shikimate. These substances are used to protect plants against herbivores, as well as to attract pollinators [89].

In the previously mentioned Iranian Borage study, the change in the essential oil formation under Se application was also examined [71]. The main components of its oils (more than 60%) are α-pinene and δ-cadinene. During foliar treatment with Na_2_SeO_3_, a gradual increase in the content of both α-pinene and δ-cadinene is observed with increasing Se concentration. However, when Na_2_SeO_4_ is added, the content of these isoprenoids first increases and then noticeably decreases [71].

Another study compared the yield of essential oil extracted from *Ocimum basilicum* L. plants depending on the treatment, such as different levels of Se in the nutrient medium and the methods of its application [90]. The results showed that the addition of selenium in the form of Na_2_SeO_4_ to the nutrient medium had a greater impact on the yield of essential oils than the foliar treatment. However, in both cases, an increase in the yield was observed with an increase in the Se concentration from 0 to 10 μM [90]. One more study examined the effect of selenium supplementation on the essential oil formation in *Ocimum basilicum* L. plants in the field conditions. The plant varieties were sprayed with Na_2_SeO_4_ (50 mg Se m^−2^) and then were used for water distillation of essential oils. Interestingly, there were no significant differences between control and treated plants [91]. Although, when considering the results of a similar study by the same authors [92], a clear difference in the yield of essential oils is found. For instance, at the first stage of the Red Rubin sampling, an increase (when using 25 mg m^−2^ of Se) in the oil content is observed, followed by a decrease with a higher selenium level (50 mg m^−2^). At the same time, there is a gradual increase in the oil content for the Dark Green variety. However, at the second stage of Red Rubin sampling, the impact of selenium remains causing a decrease in the Dark Green’s oil yield [92].

When studying the effect of selenium on the composition of essential oils isolated from *Melissa officinalis*, it was shown that the addition of 5 μM of Na_2_SeO_3_ leads to an increased formation of citral, z-citral, and geranyl acetate. That said, an increase in the content of caryophyllene and its oxide was observed at relatively low Se concentrations (0.2 μM). The authors explain this difference by the fact that citral, z-citral, and geranyl acetate are the main components of *Melissa officinalis* essential oils and have antioxidant properties. When exposed to a high concentration of selenium, plants experience oxidative stress, and these compounds are necessary to maintain the redox potential of cells [93]. These findings appear to be consistent and confirm the results of similar studies on the relationship between the level of applied selenium (regardless of the form and method of application) and the final yield of essential oil.

The data on the effect of selenium on the essential oil accumulation in plants are summarized in Table 2.

#### 3.2.2. Carotenoids

Carotenoids are tetraterpene pigments of yellow-to-purple colour. Carotenoids include carotenes (actually hydrocarbons) and xanthophylls (functional derivatives containing oxygen). Plants synthesize carotenoids via both the mevalonate and non-mevalonate pathways of isoprenoid biosynthesis. These compounds perform a number of physiologically important functions other than the photosynthetic function. Such “auxiliary” functions include but are not limited to photoprotective, attractant, antioxidant, as well as phytohormones production [101].

The function of selenium in the formation and accumulation of carotenoids does not seem obvious at first, but the research presented below highlights its fundamental role in carotenoid level modulation. For example, the effect of selenium on the content of carotene and xanthophyll in the seedlings of rice *Oryza sativa* L. of two varieties (Satabdi и Khitish) was studied [95]. The treatment of seedlings with Na_2_SeO_4_ at relatively low concentrations (<2 µM) resulted in an increase in the content of pigments (cv. Satabdi), while the use of a more concentrated Na_2_SeO_4_ solution led to their gradual decrease in both varieties [94] (Table 2). A decrease in the level of β-carotene (from 4.1 to 1.96 mg g^−1^) was also established in the study of *Solanum lycopersicu* plants treated with a solution of selenate (Na_2_SeO_4_ 1 mg L^−1^), while the levels of lycopene and lutein did not change significantly [96]. It should be noted that the effect of selenium on the accumulation of carotenoids is determined not only by the plant species or Se concentration, but also by the selected form of selenium. For example, it was shown that the treatment of the same plant seedlings with selenite (20 mg L^−1^) caused an increase in the content of carotenoids compared to control plants [95]. A study dedicated to the effect of selenium on the accumulation of carotenoids in the leaves of *Lycium chinense* also demonstrated that the introduction of selenium into the nutrient medium in the form of Na_2_SeO_3_ (from 0.01 to 0.05 g kg^−1^) led to an increase in the content of carotenoids by 200–400% [97].

The data on the effect of selenium on gene expression and the activity of key enzymes of carotenoid biosynthesis is also extremely controversial. Previously, using the model plants of *Arabidopsis thaliana*, it was shown that in the presence of 10 mmol Na_2_SeO_4_, the expression of the gene that controls the key enzyme of carotenoid synthesis, phytoensynthase, is suppressed, resulting in a decrease in the content of lutein and, as a consequence, a decrease in the total carotenoid content [85]. On the other hand, research with a different outcome should be also considered. A field study examining the effect of selenium on the chemical composition of *Zea mays* L. showed that during selenite fertigation (Na_2_SeO_3_ 200 g ha^−1^), the content of β-carotene did not change significantly, while the content of lutein and zeaxanthin increased compared to control plants. The authors attribute this increase to the probable ability of selenium to enhance the expression of phytoensynthase genes in corn plants [98,99]. At the same time, the authors attribute the insignificant change in β-carotene level to the fact that its content is primarily based on the ontogenetic development of the plant and the stage of fruit/grain maturity [96,99].

The effects of selenium on carotenoid synthesis were also shown in large-scale field studies. When the soil was treated with selenium dioxide (25 μM) in *Brassica rapa* ssp. *rapa*, a decrease in lutein content was observed [100]. The evaluation of the expression profile confirmed that the selenium treatment led to a significant increase in the expression level of a number of genes (*CYE*, *CXB*, *NCED*), the protein products of which are responsible for the biosynthesis of carotenoids [100].

### 3.3. Phenolic Compounds

Phenolic compounds are a large group of plant secondary metabolites, which include hydroxycinnamic acids, flavonoids, stilbenes, tannins, lignins, and lignans. They can be biosynthesized either by the shikimate or acetate-malonate pathway. The variety of structures explains the wide range of functions they perform in the plant body. Some of them are involved in mechanical support, others protect plants from excessive ultraviolet radiation or moisture loss. Other notable functions of phenolics include attracting pollinators and protecting against pathogens [102]. Phenolic compounds are highly labile substances, the level of which can change significantly along with fluctuations in plant growth conditions. That includes the presence of selenium, which impacts not only the phenolic content but also the phenolic profile of plants.

For example, selenate treatment was shown to increase the content of hydroxycinnamic acids in basil leaves (*Ocimum basilicum* L.). Moreover, foliar treatment had a significant effect on their accumulation: at 10 μM Se, the content of hydroxycinnamic acids was 1.6 times higher than in control plants. However, when considering the effect on the accumulation of anthocyanins, a different trend is observed. The medium supplementation had the greatest effect on the anthocyanin accumulation, whereas foliar treatment did not significantly change its level. It should be noted, that according to the data presented, the type of treatment and the concentration of selenium did not significantly affect the content of flavonoids in the leaves of *Ocimum basilicum* L. [90] (Table 3). This study also showed that plants treated with a 10 μM Se solution (regardless of the application technique) expressed the maximum total content of phenolic compounds [90].

Nutrient medium supplementation of *Oryza sativa* L. cv. Chainat 1 seedlings led to a change in the level of the studied phenolic compounds as well. For instance, when treated with Na_2_SeO_3_ (10 and 20 mg Se L^−1^), the plants expressed the highest level of the total content of phenolics. However, as selenium concentration in the nutrient medium increased to 40 mg Se L^−1^, the content of phenolic compounds decreased and became indistinguishable from the level in the control group plants. In addition, this study examined the activity of phenylalanine ammonia-lyase, a key enzyme in the phenylpropanoid biosynthetic pathway. The authors showed that the content of phenolic compounds directly correlates with the activity of this enzyme. However, the maximum activity of the enzyme is observed at a selenium concentration of 20 mg L^−1^, and the greatest content of polyphenols is observed at a selenium concentration of 10 mg L^−1^. Researchers attribute this difference to the significant use of phenolic compounds due to their antioxidant properties as plant growth intensifies [95].

A similar effect of selenium was also observed with *Spinacia oleracea* L. plants. The authors showed that selenite enrichment of the medium tended to increase the total content of phenolic compounds in plant stems. The highest content was found at 10 mg Se L^−1^ (66% increase). Interestingly, the reverse trend was observed for the roots: an increase in the concentration of selenium led to a decrease in the amount of phenolic compounds. However, exposure to low concentrations of selenium (1–4 mg Se L^−1^) led to an increase in the total phenolics content by about 2 times [103].

Another field study addressed the effect of selenium on the biosynthesis of anthocyanins in wheat plants of the 202w17 variety (purple-grain). Selenium at a concentration of 37.5 g ha^−1^ (as Se^4+^) was applied in two different ways: soil supplementation and spraying. It was found that soil supplementation has the greatest effect on the content of anthocyanins compared to spraying (42 and 26% increase, respectively, compared with the control samples). The study also showed a positive correlation between the content of anthocyanins and the total protein and free amino acid content [104].

A study by Linling et al. [109] showed that selenium can influence the level of expression of genes encoding key enzymes involved in anthocyanin pigment biosynthesis pathways. For example, spraying the leaves of *Ginkgo biloba* with a solution of sodium selenite (10 mg mL^−1^), led to an observable increase in the level of expression of genes encoding chalcone synthase, flavonol synthase, flavanoid-O-methyltransferase, phenylalanine ammonia-lyase. Although, supplementing the roots with sodium selenite did not cause a significant increase in gene expression [109].

In another study, it was shown that the addition of selenium (0.1 mmol L^−1^) to the soil of *Brassica oleracea* L. var. *italica* resulted in a significant increase in flavonoid levels. However, a further increase in concentration (up to 0.4 mmol L^−1^) led to a sharp decrease in the content of flavonoids in the experimental plants, associated with a sharp increase in the content of glycosinolates. The authors explain this phenomenon by the fact that although glucosinolates and phenylpropanoids are biosynthesized by different biochemical pathways, there is a cross-interaction between their biosynthetic pathways. This interaction manifests itself in the accumulation of glucosinolate intermediates, which limit the formation of phenylpropanoids. Analysis of the metabolome and transcriptome confirms this assumption [76].

## 4. Possible Pathways of Regulating the Biosynthesis of Secondary Metabolites via Selenium Supplementation

### 4.1. Selenium Impact on Primary Sulphur and Nitrogen Metabolisms of Plants

Since selenium and sulfur compete for the same metabolic pathways, selenium accumulation has an effect on gene expression similar to sulfur deficiency. The presence of Se affects the expression of key genes encoding S-transporters and enzymes that regulate S-metabolism. The absorption and assimilation of sulfur by plants are coordinated with the absorption and assimilation of nitrogen. Thus, by influencing sulfur metabolism, selenium also affects nitrogen metabolism. In addition, selenium can interfere with nitrogen metabolism by interfering with the absorption of molybdenum, which is a cofactor for nitrate reductase (an enzyme responsible for nitrogen assimilation). The change in N assimilation has serious consequences for the synthesis of all nitrogen-containing metabolites, including amino acids, proteins, and nucleotides, as well as secondary metabolites, including phytohormones, phenylpropanoids, alkaloids, glucosinolates, phytoalexins, etc. The mechanisms behind the selenium effect on sulfur and nitrogen metabolism are described in more detail by White [2], and Schiavon et al. [110].

### 4.2. Selenium Impact on Hormonal Regulation in Plants

An essential factor in the development, primary and secondary metabolism of plants is hormonal regulation. Unlike animals, plants appear to use fewer hormones. Nine plant hormones are known: auxin, cytokinins, ethylene, gibberellins, abscisic acid, brassinosteroids, jasmonate, salicylic acid, and strigolactones; they are found in all land plants [111]. Studies on the regulation of the biosynthesis of various classes of secondary metabolites showed that various hormone-signaling pathways are involved in the regulation. Examples of such pathways are the induction of flavonoid biosynthesis by abscisic acid, jasmonate, or cytokinins, and its repression by gibberellic acid, ethylene, or brassinosteroids [112].

Selenium compounds can modulate the signal from plant hormonal stimuli significantly. For example, selenium was shown to inhibit the biosynthesis of auxin and ethylene in rice seedlings, changing their primary metabolism and the architecture of the root [113]. The mechanism of such an effect is not fully understood. It is believed that Se induces changes in the interactions between auxin and ethylene to mediate the control of primary and lateral root development in rice. It was previously shown that Se at low concentrations can stimulate primary root elongation by triggering the biosynthesis and transport of auxins in tobacco (*Nicotiana tabacum*) [114]. However, with an increase in selenium concentration to 40 μM in experimental Arabidopsis plants, a decrease in the expression of the auxin-sensitive *DR5::GUS* gene was observed, while the expression of the cytokinin-induced *ARR5::GUS* gene and the *ACS8::GUS* gene, responsible for ethylene biosynthesis, increased instead [115]. Data on the effect of selenium compounds on the expression of genes for auxin-sensitive proteins, including the transporters of auxin itself, also look contradictory. Therefore, selenium makes a significant contribution to the distortion of the normal signal from auxin and ethylene, and also distorts the coordination between these two regulatory systems.

In addition, the relationship between selenium metabolism and hormonal systems is confirmed by studies of the mechanisms behind the acquisition of selenium resistance in some plants. In particular, in a transcriptome study conducted to identify selenate-responsive genes, the expression of many ethylene and/or jasmonic acid (JA)-responsive genes was induced by selenate treatment [116,117]. Arabidopsis plants treated with selenite also showed an accumulation of salicylic acid [116].

Thus, selenium can indirectly affect the overall metabolism (both primary and secondary) of plants through the modulation of the hormonal signal. It should also be noted, that selenium affects the root architecture of the plant, interfering with the uptake of other nutrients from the soil medium.

### 4.3. Selenium Impact on the Participants of Plant Redox Metabolism

One of the mechanisms affecting the biosynthesis of secondary metabolites in plants is redox regulation. It is known that ROS act as secondary messengers and actively interact with other signaling molecules, such as mitogen-activated protein kinases (MAPKs), phosphatases, calcium (Ca^2+^), and activator proteins [118]. Then, the signals are transmitted by a number of intermediaries (such as JAZ, MYC2, and EIN3) to downstream transcription factors. These transcription factors bind directly to cis-elements of gene promoters to regulate gene expression and subsequent biosynthesis of secondary metabolites [119]. Some genes, the protein products of which are involved in the synthesis of secondary metabolites, and transcription factors that regulate secondary metabolism, are sensitive to the concentration of ROS and the redox status of the cell. For example, among the genes that respond to changes in the concentration of H_2_O_2_ in cells, there are a relatively large number of genes encoding UDP-glucuronosyl/UDP-glucosyltransferases (UGT) [120]. UGT catalyzes the glucose transfer (as UDP-glucose) to a wide range of acceptor molecules, including plant hormones and all major classes of plant secondary metabolites, such as hydroxycinnamic acids, flavonoids, anthocyanins, glucosinolates, cyanogenic glycosides, and others [121]. Redox control is thought to influence MYB protein activity because of the presence of a pair of conserved Cys residues. These residues are included in the R2-MYB motif of most proteins of the 3R- and R2R3-MYB classes, which are involved in the regulation of the biosynthesis of flavonoids and glucosinolates. When oxidized, these two Cys-residues form an intramolecular S-S bond in the protein, which significantly changes the structure of the MYB domain, preventing DNA binding [122]. Selenium can affect the redox metabolism of plants and, as a result, the biosynthesis of secondary metabolites in several ways.

First, selenium, or rather some of its organic derivatives, can act as antioxidants, reacting with ROS directly. Such antioxidant effect was shown for selenocysteine and selenomethione, which can be explained by the nucleophilic properties of ionized selenol (RSe^−^, prevailing over the neutral form at physiological pH values) and the ease of oxidation of these selenium-containing amino acids [123]. In addition, high antioxidant activity, in particular the ability to neutralize O2^•−^, DPPH^•^, ABTS^+•^ radicals, was established for selenium-containing proteins and peptides isolated from plants [124,125]. Studies of selenoproteins/selenopeptides showed that they contribute more to antioxidant activity than conventional proteins. Antioxidant abilities are also correlated with increased content of Se in proteins [124]. For example, a selenopeptide with the SeMet-Pro-Ser sequence showed higher antioxidant activity compared to the Met-Pro-Ser peptide [126]. Studies comparing the antioxidant activity of SeMet and Met showed that these amino acids react with hydroxyl radicals with the same mechanism. However, SeMet’s lower redox potential allows it to oxidize more easily than Met [127]. Thus, being present in plant tissues, organoselenium compounds are capable of exerting a direct antioxidant effect, thereby changing the redox status and regulating the biosynthesis of secondary metabolites.

Second, selenium can influence the metabolism of glutathione and the enzymes involved in maintaining redox homeostasis in cells. Genes and proteins associated with glutathione metabolism play an important role in plant selenium assimilation and tolerance [128]. However, the results of studies on the effect of selenium on the level of glutathione and the expression of genes involved in its metabolism vary greatly depending on the type of plant, the form, and the concentration of selenium. For example, transcriptome analysis of *Cardamine violifolia* plants treated with selenium (as selenate; 0, 0.25, 1.0, 4.0, 16.0, 64.0, 256.0 mg L^−1^) revealed a decrease in the expression of genes related to glutathione metabolism, such as genes encoding glutathione gamma-glutamyl-cysteinyl-transferase 1, glutathione S-transferase F12, glutamate-glyoxylate aminotransferase 1, and glutamine amidotransferase class-I, which ultimately led to a decrease in glutathione levels in cells [129]. At the same time, the results of transcriptomic analysis of tea plants treated with selenium (as selenite, 0, 0.015, 0.025, 0.050, 0.100, 0.200, 0.400 mM) showed that the roots had significantly higher expression of 121 genes encoding glutathione S-transferase (GST), glutathione synthetase, glutathione peroxidase, glutathione reductase and glutaredoxin [117]. Among the enzymes listed above, researchers pay particular attention to the relationship between selenium and glutathione peroxidase. It is known that in animals this enzyme contains selenium in its active center and, as a result, its activity is directly related to the presence of this microelement. Generally, the increase of GPs activity upon the addition of Se was observed in multiple experiments, which indicates the decisive role of this enzyme in counteracting oxidative stress in plants [68]. Moreover, the activity of other antioxidant enzymes, such as superoxide dismutase (SOD), ascorbate peroxidase (APX), catalase (CAT), and glutathione reductase (GR), was also found to increase after the addition of selenium in various plant species [2].

Third, selenium and its derivatives, on the contrary, can act as prooxidants themselves, provoking oxidative stress in plant cells and thereby triggering protective mechanisms, including the biosynthesis of some secondary metabolites, such as phenolics. Despite the fact that this assumption is in some contradiction with the previously described antioxidant properties of organoselenium compounds, there is a sufficient amount of experimental data confirming the link between the treatment of plants with selenium, the following development of oxidative stress in them, and the resulting changes in biosynthesis of secondary metabolites. This property of selenium is manifested very clearly when it is applied at high concentrations and/or in experiments with non-accumulators. For example, in studies on kale sprouts, the increased lutein biosynthesis was associated with the need to maintain low levels of ROS and prevent lipid peroxidation in the thylakoid membrane, which is triggered at high levels of selenium [130]. Similar results were obtained for lettuce plants, which showed the maximum content of phenolic compounds, flavonoids, and ascorbic acid when grown at the highest selenium concentration, at which the plants developed oxidative stress [131]. An enhanced biosynthesis of phenylpropanoids as a result of selenium-induced changes in the H_2_O_2_ and/or NO concentration was also observed in peanut [132], rice [133], wheat [106], and green basil seedlings [134]. It is believed that what causes ROS accumulation and oxidative stress in plants treated with selenium is a disturbance of glutathione metabolism, leading to a decrease in its content [37], abnormal incorporation of Se into proteins and, as a result, disruption of their functions [135], as well as selenium-induced spontaneous dismutation of the superoxide radical (O_2_^•−^) into H_2_O_2_ [136].

Some authors note not only selenium-induced oxidative stress but also nitrosative stress (stress that stimulates the production of reactive forms of nitrogen, in particular, NO and peroxynitrite) [137]. It is also known that NO is an important signaling molecule and, together with salicylic acid, jasmonic acid, and ROS, it is involved in the regulation of the biosynthesis of secondary metabolites in plants [138]. Thus, disruption of homeostasis of reactive nitrogen species under the action of selenium can also lead to changes in secondary metabolism.

### 4.4. Selenium Impact on the Expression of Genes for the Biosynthesis of Secondary Metabolites and Transcription Factors

In Section 3, when considering the effect of selenium on the accumulation of secondary metabolites, it was mentioned that the treatment of various plant species with selenium led to a change in the expression of genes involved in the biosynthesis of secondary metabolites.

For example, in a study on the effect of selenium treatment on the biosynthesis of glucosinolates in broccoli, selenium was shown to significantly reduce the expression of *BCAT4* and *MAM1*, which are involved in chain elongation; *CYP79B2*, *CYP79F1*, *CYP83B1*, and *CYP83A1*, which are involved in the formation of the core glucosinolate structure; and *UGT74B1* and *FMO2*, which are involved in secondary modifications. In addition, exposure to selenate dramatically suppressed genes encoding the transcription factors MYB28 and MYB34, which regulate aliphatic- and indole-glucosinolate synthesis [73]. At the same time, data on the effect of selenium on the expression of genes for the biosynthesis of glucosinolates are contradictory even in experiments on the same plant species—broccoli. Mao et al. [75] found no significant changes in the expression of *BCAT3, MAM1, CYP79F1, CYP83A1, SUR1*, *UGT74C1,* and *MYB76* when plants were treated with selenium. However, a significant increase in the expression of *MY*, the gene encoding myrosinase, and a decrease in the expression of *Myb28* were found [75]. In a study on radish plants, a tissue-specific transcriptome reaction to the treatment of plants with selenium was demonstrated. The leaves showed a decrease in the expression of transcription factor *Myb28*, glucosyltransferase *Ugt74b1*, *MY,* and an increase in the expression of the sulfate transporter gene *Sultr2;1* when treated with selenium at a concentration of 40 μM. Changes in the expression of other studied genes were not significant. The roots, on the contrary, showed a more pronounced reaction at the transcriptome level. Expression of the genes encoding Sultr2;1 Atps1, Myb28, Ugt74b1, Myr, and Eps was higher in plants treated with selenium, especially at high concentrations (40 μM) [78].

Other experimental studies showed that selenium also affects the expression of carotenoid biosynthesis genes. In particular, an increase in the transcriptional level of the *CYE* (phytoene synthase), *CXB*, and *NCED* (9-cis-epoxycarotenoid dioxygenase) genes in rapeseed leaves and *CYE* in corn was found [99,100]. In addition, in Arabidopsis plants, the gene controlling phytoene synthase (PSY) was suppressed by the presence of Se [85].

There are more experimental data confirming the effect of selenium on the expression of key genes for the biosynthesis of phenolic compounds. For example, in a study based on the treatment of *Ginkgo biloba* leaves with a solution of sodium selenite, an increase in the expression level of the *PAL*, *CHS*, *FLS*, and *FOMT* genes was observed. Expression of *MYB1* and *MYB2* (transcription factors that play a key role in flavonoid biosynthesis) was also higher when plants were treated with Se. These data indicate that *MYB1* and *MYB2* are involved in the positive regulation of flavonoid accumulation upon Se treatment [109].

Rao et al. (2021), using a combined transcriptomic and metabolomic analysis, found that the sulfate transporter genes *Sultr3;1*, *Sultr4;2* and some cytochrome P450 genes (e.g., *CYP71B21*, *CYP72C1*, and *CYP81F1*) may be involved in the regulation of the biosynthesis of glucosinolates and flavonoids in broccoli. It is known that sulfate transporters homologous to *AtSULTR4;1* and *AtSULTR4;2* are present in the tonoplast of plant cells and catalyzes the movement of selenate from/to the vacuole [50]. Expression of the *AtSULTR4;1* and *AtSULTR4;2* genes increases both with an excess of Se and with a deficiency of S in the medium [139]. It is believed that the sulfate transporter AtSULTR3;1 is localized in the chloroplast membrane and can probably catalyze the transport of selenate into plastids [56]. It is in plastids that selenate is converted into organic forms of selenium, which later become parts of the metabolic pathways of plants. Thus, the effect of selenium on the secondary metabolite level may be mediated by changes in the activity of sulfate transporters and the involvement of organic forms of selenium in metabolism.

Another possible way selenium affects secondary metabolism may be associated with plant epigenetic regulatory strategies, in particular DNA methylation. It is known that DNA methylation can be considered a precise protective mechanism for the regulation of gene expression. Generally, hypermethylation correlates with gene silencing, while hypomethylation is associated with active transcription. One of the triggers for methylation is reactive oxygen species. It was shown that the treatment of plants with selenium can lead to both an increase and a decrease in the level of ROS, depending on the selenium concentration and type of plant [37]. This way, by changing the concentration of ROS in cells, selenium can indirectly affect the degree of DNA methylation, which ultimately leads to changes in gene expression, including those responsible for the biosynthesis of secondary metabolites [99,140].

**Figure 3:**
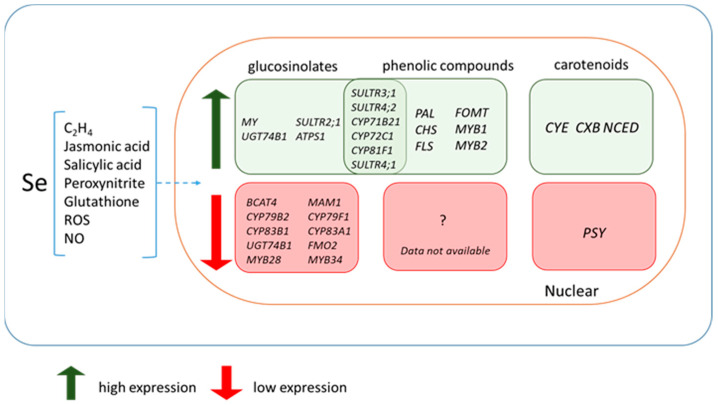
Effect of Se on the expression of genes and transcription factors involved in the secondary metabolism biosynthesis.

Thus, changes in gene expression of selenium-treated plants can be mediated both by changes in their primary metabolism and hormonal regulation and by the effect of selenium on redox metabolism in cells (Figure 3).

## 5. Conclusions and Research Perspectives

Molecular research, including omics technologies such as transcriptomics-, proteomics-, and metabolomics-based methods, made it possible to achieve significant success in the study of plant metabolism and the role of selenium in it. To date, a fairly large array of experimental data was accumulated, showing the effect of selenium not only on the primary metabolism of plants but also on the accumulation of various secondary metabolites (alkaloids, cyanogenic glucosides, glucosinolates, terpenes, phenolic compounds) in various plant species. However, these data are extremely contradictory and often difficult to compare, since studies are carried out on different types of plants, different stages of ontogenesis, and growing substrates, with different methods of treatment with selenium, in different forms and at different concentrations. This leads to the fact that some studies show a stimulating effect of selenium on the accumulation of metabolites, in others—an inhibitory effect, and in others—no effect at all. This heterogeneity of approaches has not yet made it possible to draw an unambiguous conclusion about the significance of selenium as a nutrient. This problem can be solved, for example, by conducting experiments under the same conditions, but with plants characterized by a different ability to accumulate selenium and, accordingly, a different intensity of selenium participation in primary and secondary metabolism.

In this review article, the following aspects were identified as possible ways of regulating plant secondary metabolism by selenium-supplementation: changes occurring in primary S/N metabolism, hormonal regulation, redox metabolism, as well as at the transcriptomic level of secondary metabolite biosynthesis. However, most likely, these are not the only regulatory mechanisms in which selenium is involved and which cause changes in the accumulation of secondary metabolites. We attempted to evaluate each of the known pathways separately. Secondary metabolite levels can be influenced by a large group of factors; therefore, only the correct setting of the experiment would allow a targeted assessment of the role of Se.

The relative unification of experimental approaches, as well as the later expansion of the testing range, would make it possible to assess both the adaptogenic and metabolism-enhancing potential of Se and the toxic nature of this element. This approach can provide more precise results even at the level of field experiments. In addition, Se has the potential as an agent for biofortification or phytofortification that improves the nutritional quality of food and feed, however, only a few crops have been studied in this aspect. The study of Se-mediated metabolic pathways, the involvement of various forms of selenium in signaling pathways, including the search for intermediates (messengers, activator proteins, transcription factors) that can directly bind to selenium or its derivatives can provide a deeper understanding of existing data and make a certain contribution to understanding the actual role of Se in the growth, development, and productivity of plant organisms.

## Figures and Tables

**Table 1:** Effect of Se application on the accumulation of alkaloids, cyanogenic glycosides, and glucosinolates.

Plant/Cultivars/Plant Organ	Se Application (Type of Treatment, Form, Concentration)	Secondary Metabolites	Changes	Reference
Alkaloids				
*Fragaria* × *ananassa* fruits	nutrient solution (hydroponics), Na_2_SeO_4_, 10 and 100 μM	gramine	↑ ^1^ 100 μM	[70]
*Echiuma moenum* Fisch. & Mey. (Iranian borage) leaves, petals	foliar application, Na_2_SeO_3_/Na_2_SeO_4_ 2, 4, 8 and 16 mg L^−1^	total alkaloids content	↑ 4 mg L^−1^ Na_2_SeO_4_	[71]
*Lupinus albus* L. seeds	foliar application, Na_2_SeO_3_, 0.01%	total alkaloids content	↓	[72]
Cyanogenic Glycosides				
*Fragaria* × *ananassa* fruits	nutrient solution (hydroponics), Na_2_SeO_4_, 10 and 100 μM	3,4-dihydroxymandelonitrile-β-glucoside	↓ 10 μM	[70]
Glucosinolates				
*Brassica oleracea* L. var. *italic* (broccoli) sprouts, young leaves, florets	nutrient solution or soil irrigation, Na_2_SeO_4_, 25 μM	total glucosinolate content	NS (sprouts) ↓ (leaves and florets)	[73]
		glucoraphanin	NS (sprouts) ↓ (leaves and florets)	
		hydroxyglucobrassicin	NS (sprouts, leaves, and florets)	
		glucobrassicin	NS (sprouts, leaves, and florets)	
		methoxyglucobrassicin	NS (sprouts, leaves, and florets)	
		neoglucobrassicin	NS (sprouts) ↓ (leaves and florets)	
		glucoerucin	NS (sprouts, leaves, and florets—n.d.)	
*Brassica oleracea* L. var. *italic* (broccoli) three cultivars (Fenglei 60, Wenxing 90, and Shenglv 120) sprouts	spraying, Na_2_SeO_3/_Na_2_SeO_4_, 100 μmol L^−1^	total glucosinolate content	NS (Fenglei 60, Shenglv 120) ↑ Na_2_SeO_4_ (Wenxing 90)	[74]
		sulforaphane	↑ Na_2_SeO_3_ (Fenglei 60, Wenxing 90) ↑ Na_2_SeO_4_ (Shenglv 120)	
*Brassica oleracea* L. var. *italic* (broccoli) florets	foliar application, Na_2_SeO_3_, (S mM/Se μM: 1/0, 1/50, 1/100, 1/150, 4/0, 4/50, 4/100, and 4/150)	total glucosinolate content	↓ 1/150 (S mM/Se μM)	[75]
		glucoiberin	↑ 1/100–150 and 4/150 (S mM/Se μM)	
		glucoraphanin	↓ 1/150 (S mM/Se μM)	
		gluconapin	NS	
		glucoibervirin	↑ 1/50–100 (S mM/Se μM) ↓ 4/50–150 (S mM/Se μM)	
		glucoerucin	↑ 4/50–150 (S mM/Se μM)	
		n-hexyl-glucosinolates	NS	
		4-hydroxyglucobrassicin	↑ 1/150 and 4/50–150 (S mM/Se μM)	
		glucobrassicin	↓ 1/150 (S mM/Se μM)	
		4-methoxyglucobrassicin	↓ 1/100–150 (S mM/Se μM)	
		neoglcobrassincin	↓ 1/50–150 (S mM/Se μM)	
		gluconasturtiin	↓ 1/50–150 (S mM/Se μM) ↑ 4/50 and 4/150 (S mM/Se μM)	
		sulforaphane	↑ 1/100 and 4/50–100 (S mM/Se μM)	
*Brassica oleracea* L. var. *italic* (broccoli) florets	soil irrigation, Na_2_SeO_4,_ 0.1, 0.2, 0.4, 0.8, and 1.6 mmol L^−1^	total glucosinolate content	↑ 0.4 mmol/L	[76]
*Brassica oleracea* L. var. *italic* (broccoli) florets	soil irrigation, Se yeast/Na_2_SeO_3,_ 0.1, 0.2, 0.4, 0.8 and 1.6 mmol L^−1^	total glucosinolate content	↑	[77]
*Raphanus sativus* (radish) leaves, roots	foliar, Na_2_SeO_4_, 5, 10, and 20 mg Se per plant nutrient solution (hydroponics), Na_2_SeO_4_, 5, 10, 20, or 40 μM	total glucosinolate content	↑ (foliar, 20 mg plant^−1^, roots) ↓ (nutrient solution, 40 μM, leaves)	[78]
		glucoraphanin	↑ (foliar, 20 mg plant^−1^, roots)	
		glucoraphasatin	↓ (foliar, 10–20 mg plant^−1^, leaves; nutrient solution, 5–40 μM, leaves) ↑ (foliar, 20 mg plant^−1^, roots)	
		glucobrassicin	↑ (foliar, 5 mg plant^−1^, leaves) ↓ (nutrient solution, 5–40 μM, leaves)	
		neoglucobrassicin	↑ (foliar, 20 mg plant^−1^, leaves, roots)	
		dimeric-4 mercaptobutyl (DMB-GLS)	↑ (foliar, 20 mg plant^−1^, roots; nutrient solution, 20 μM, roots) ↓ (foliar, 10 mg plant^−1^, roots)	
*Eruca Sativa* Mill. (salad rocket) leaves	nutrient solution (hydroponics), Na_2_SeO_4,_ 5, 10, 20, 40 μM	total glucosinolate content	↓ 20–40 μM	[79]
		glucoraphanin	↓ 20–40 μM	
		glucocheirolin	NS	
		glucoerucin	↓ 20 μM	
		DMB-GLS	↓ 10–40 μM	
		glucosativin	NS	
		neoglucobrassicin	NS	
*Diplotaxis tenuifolia* (wild rocket) leaves	nutrient solution (hydroponics), Na_2_SeO_4,_ 5, 10, 20, 40 μM	total glucosinolate content	↓ 20–40 μM	[79]
		glucoraphanin	↓ 20–40 μM	
		glucocheirolin	NS	
		glucoerucin	NS	
		DMB-GLS	NS	
		glucosativin	NS	
		neoglucobrassicin	↓ 20 μM	

^1^ The arrows ↑ and ↓ indicate increases and decreases in secondary metabolite content, respectively. NS—not significant changes.

**Table 2:** Effect of Se application on the accumulation of terpenes.

Plant/Cultivars/Plant Organ	Se Application (Type of Treatment, Form, Concentration)	Secondary Metabolites	Changes	Reference
Essential oils/Mono- and Sesquiterpenes				
*Echiuma moenum* Fisch. & Mey. (Iranian borage) leaves, petals	foliar application, Na_2_SeO_3_/Na_2_SeO_4_, 2, 4, 8 and 16 mg L^−1^	β-cadinene	↑ ^1^ (2–16 mg L^−1^ Na_2_SeO_3_) ↑ (2–8 mg L^−1^ Na_2_SeO_4_)	[71]
		α-pinene	↑ (2–16 mg L^−1^ Na_2_SeO_3_) ↑ (2–8 mg L^−1^ Na_2_SeO_4_)	
*Ocimum basilicum* L. (basil) leaves	foliar application/nutrient solution (hydroponics), Na_2_SeO_4_, 2.0, 5.0, and 10.0 μM	essential oil content	↑ (2–10 μM, nutrient solution) ↑ (5–10 μM, foliar application)	[90]
*Ocimum basilicum* L. (basil) three cultivars (Red Rubin, Purple Ruffles, Dark Green) shoots	soil fertilization, Na_2_SeO_4,_ 50 mg Se m^−2^	essential oil content	NS	[91]
*Ocimum tenuiflorum* L. (Tulsi basil) shoots	soil fertilization, Na_2_SeO_4_, 50 mg Se m^−2^	essential oil content	NS	[91]
*Ocimum basilicum* L. (basil) two cultivars (Red Rubin, Dark Green), two harvest shoots	soil fertilization, Na_2_SeO_4_, 25 and 50 mg Se m^−2^	essential oil content	↑ (Red Rubin 25 mg Se/m^2^) ↓ (Red Rubin 50 mg Se/m^2^) ↑ (Dark Green, 1st harvest) ↓ (Dark Green, 2nd harvest)	[92]
*Melissa officinalis* L.	nutrient solution (hydroponics), Na_2_SeO_3_, 2.0, 5.0 and 10.0 μM	citral	↓ 0.2 μM ↑ 5.0 μM	[93]
		z-citral	↓ 0.2 μM ↑ 5.0 μM	
		caryophyllene	↑ 0.2 μM ↓ 5.0 μM	
		caryophyllene oxide	↑	
		geranyl acetate	↑ (5.0 μM)	
Carotenoids				
*Oryza sativa* L. (rice) two cultivars Satabdi and Khitish seedlings	nutrient solution (hydroponics), Na_2_SeO_4_, 2, 10, 20, 50 μM	carotene	↑ 2 μM (Satabdi) ↓ 10–50 μM (Satabdi) ↓ 2–50 μM (Khitish)	[94]
		xanthophyll	↑ 2 μM (Satabdi) ↓ 10–50 μM (Satabdi) ↓ 2–50 μM (Khitish)	
*Oryza sativa* L. (rice) seedlings	nutrient solution (hydroponics), Na_2_SeO_3_, 10, 20, 30, 40 mg Se L^−1^	total carotenoids content	↑ 20 mg Se/L	[95]
*Solanum lycopersicu* L. (tomato) fruits	nutrient solution (hydroponics), Na_2_SeO_4_, 1 mg Se L^−1^	lycopene	NS	[96]
		lutein	NS	
		β-carotene	↓	
*Lycium chinense* L. leaves	perlites, Na_2_SeO_3_, 0.01, 0.02, 0.03, 0.04, and 0.05 g kg^−1^	total carotenoids content	↑	[97]
*Arabidopsis thaliana* shoots	nutrient solution (hydroponics), Na_2_SeO_4_, 10 μM	lutein	↓	[85]
		b-carotene	NS	
		violaxanthin	NS	
		neoxanthin	NS	
*Zea mays* L. (maize) grains	soil fertilization, Na_2_SeO_3_, 200 g of Se ha^−1^	lutein	↑	[98]
		zeaxanthin	↑	
		β-carotene	NS	
*Zea mays* L. (maize) grains	soil fertilization, Na_2_SeO_3_, 50 mg of Se kg^−1^	total carotenoids content	↑	[99]
*Brassica rapa* ssp. *rapa *(turnip) plant	soil irrigation, SeO_2_, 25 μM	lutein	↓	[100]

^1^ The arrows ↑ and ↓ indicate increases and decreases in secondary metabolite content, respectively. NS—not significant changes.

**Table 3:** Effect of Se application on the accumulation of phenolic compounds.

Plant/Cultivars/Plant Organ	Se Application (Type of Treatment, Form, Concentration)	Secondary Metabolites	Changes	Reference
*Ocimum basilicum* L. (basil) leaves	foliar application/nutrient solution (hydroponics), Na_2_SeO_4_, 2.0, 5.0, and 10.0 μM	the total hydroxycinnamic acid content	↑ ^1^	[90]
		total anthocyanin content	↑ (5–10 μM, nutrient solution)	
		total flavonoid content	NS	
		total phenolic compound content	↑ (2–10 μM, nutrient solution, 5–10 μM foliar application)	
*Oryza sativa* L. (rice) seedlings	nutrient solution (hydroponics), Na_2_SeO_3,_ 10, 20, 30, 40 mg Se L^−1^	total extractable phenolic content	↑ 10–20 mg Se/L	[95]
*Spinacia oleracea* L. (spinach) shoots, roots	nutrient solution (hydroponics), Na_2_SeO_3_, 1, 2, 4, 6 and 10 mg L^−1^	total phenolic compound content	↑ 4–10 mg Se/L (shoots) ↑ 1–4 mg Se/L (roots)	[103]
Wheat two cultivars (202w17 and Shannong 129) grain	soil/foliar applications, Se^4+^, 50 mg kg^−1^/50 mg Se L^−1^	total anthocyanins	↑ (cv. 202w17) NS (Shannong 129)	[104]
*Brassica oleracea* L. var. *italic* (broccoli) florets	soil irrigation, Na_2_SeO_4_, 0.1, 0.2, 0.4, 0.8, and 1.6 mmol L^−1^	flavonoid content	↑ 0.1 mmol/L ↓ 0.4 mmol/L	[73]
*Malus domestica* two cultivars (Golden Delicious, Jonagold) fruits	spraying, Na_2_SeO_3_/Na_2_SeO_4_, 0.15 kg Se per hectare and meter canopy height	total phenolic content	NS	[105]
		chlorogenic acid	NS	
		epicatechin	NS	
		procyanidin trimer	NS	
		caffeoyl glucoside	NS	
*Raphanus sativus* (radish) leaves, roots	foliar, Na_2_SeO_4_, 5, 10, and 20 mg Se per plant nutrient solution (hydroponics), Na_2_SeO_4_, 5, 10, 20, or 40 μM	kaempferol-3-glucoside	↓	[84]
		kaempferol-7-O-rhamnoside	NS	
		caffeic acid	↑ (5–10 mg per plant) ↓ (20 mg per plant)	
		kaempferol-3-rhamnosil glucoside	↑ (20 mg per plant, leaves) NS (roots)	
		kaempferol-3-O-arabinoside-7-O-rhamnoside	↑ (20 mg per plant, leaves)	
		kaempferol-3,7-dirhamnoside	↑	
		coumaric acid	NS (leaves) ↓ (roots)	
		sinapic acid	↑ (5 mg per plant, leaves)	
		ferulic acid	↓ (10–20 mg per plant, leaves, roots)	
		feruilmalate	↓ (20 mg per plant)	
		sinapoilmalate	↓ (20 mg per plant)	
*Triticum aestivum* L. (winter wheat) microgreens	nutrient solution (hydroponics), Na_2_SeO_3_, 0.125, 0.25, 0.50, and 1.00 mg L^−1^	total phenolic content	↑ (0.25–0.5 mg/L)	[106]
		total flavonoid content	↑ (0.5 mg/L)	
		anthocyanin content	↑ (0.25 mg/L)	
*Brassica oleracea* var. *botrytis* (cauliflower) two cultivars (Clapton, Graffiti) floret	foliar application, Na_2_SeO_4_, 0, 5, 10, 15, 20 mg L^−1^	total phenolic compounds	↑ (10–20 mg/L)	[107]
*Valerianella locusta* L. Laterr. (lamb’s lettuce) three harvest (38, 52 and 66 DAS) shoots	nutrient solution (hydroponics), Na_2_SeO_4_, 5.0, 10.0, and 20.0 µM	chlorogenic acid	↑ 5 µM	[108]
		the total hydroxycinnamic acid content	↑ 5 µM	
		total flavonoid content	↑ (10–20 µM at 38 and 52 DAS, 5 µM at 66 DAS)	
		total phenolics	↑ (5 µM at 38 and 66 DAS, 5–20 µM at 52 DAS)	

^1^ The arrows ↑ and ↓ indicate increases and decreases in secondary metabolite content, respectively. NS—not significant changes; DAS—days after sowing.

## Data Availability

Not applicable.
